# The Role of Philosophical Inquiry in Helping Students Engage in Learning

**DOI:** 10.3389/fpsyg.2020.00449

**Published:** 2020-03-24

**Authors:** Lu Leng

**Affiliations:** College of Foreign Studies, Jinan University, Guangzhou, China

**Keywords:** learning engagement, Philosophy for Children Hawaii, adolescent academic achievement, philosophical inquiry, motivation

## Abstract

Studies have characterized high school students as bored, alienated, and disconnected with their class and the learning process. In order to address this problem to improve student learning engagement, this study explores the impact of philosophical inquiry (PI) on the development of adolescents’ academic engagement and adds to the scholarly research on Philosophy for Children (P4C). In determining an appropriate and holistic approach to investigating students’ learning engagement and motivation from the perspective of psychology, this study involves multiple forms of data collection, specifically including surveys, student work, focus group interviews, classroom discussions, and reflective notes. Applying a qualitative method, this multiple case study developed a deeper understanding of the classroom contexts, conditions, discourses, tools, and practices that promote positive adolescent learning experiences. The study developed a conceptual framework of student academic engagement in a PI class and summarized reasons why the participants engaged in learning. First, students believed that maintaining a safe and positive classroom environment is a fundamental condition for learning. Second, they reported that asking questions, sharing ideas, listening attentively, thinking deeply, and making connections are the manifestations of an engaging classroom. Third, students reported that they transcended their learning experiences by living a new philosophy that was acquired in the process of the community of inquiry. The study found that PI enhanced social inclusion and active participation of the participant in the learning process.

## Introduction

Philosophical inquiry (PI), or the practice of “thinking together” and “thinking about thinking together,” is an educational approach that originates from philosophical pragmatism. It upholds that knowing is not merely an acquisition of knowledge that is external to the knower, but arises from a community of inquiry that students engage with and construct together. This pedagogical approach involves logical questioning and broad discussions among students and their teachers. The teacher is the students’ coparticipant. In the process of constructive dialog, students and their teacher clarify thinking, raise questions, record discussions, explore meanings, listen carefully, and respond to the ideas of others respectfully and non-judgmentally (Millett and Tapper, 2011). Considering the benefits of PI, the Hawai‘i State Department of Education developed a standards-based social studies course called PI that helps students understand and gain knowledge in transforming what they learn into daily practice and problem solving. In this PI course, students will build an intellectually safe place (Jackson, 2001) where students and teachers use dialog, gentle Socratic inquiry, responsible thinking, and empathy to examine questions and issues that arise from their genuine wonderings about the study of history, psychology, contemporary society, economics, political science, geography, and social interaction. During the inquiry, students learn tools for thinking philosophically, critically, and ethically across the wide range of interdisciplinary topics and issues introduced in each area of scholarship (Hawaii public schools course description catalog, 2019).

The PI course is grounded in the Philosophy for Children Hawaii (p4cHI) approach to education and helps students and teachers to create a more thoughtful, compassionate, and ethical educational experience. p4cHI is an outgrowth and unique expression of Matthew Lipman’s (1988, 2003) original Philosophy for Children (P4C) movement. p4cHI is an innovative approach to education that transforms the schooling experience by engaging students in intellectually safe communities of inquiry where students and teachers continue to develop their ability to think for themselves in responsible ways (p4cHI website, 2020). p4cHI has now become the namesake of the educational movement associated with doing philosophy with K-12 and university students in the Hawaiian islands (Miller, 2013). The PI course was piloted in the Hawaii State Department of Education (HI DOE) at Kailua High School (KHS) in the fall of 2013. This research will examine the effects of the PI course on students’ learning engagement.

## Research Purpose

The purpose of this qualitative research is to explore how a PI course that utilizes p4cHI educational approach featuring a community of inquiry, philosophical thinking, and reflection contributes to adolescents’ engagement to learning. It is hoped that the theoretical and academic engagement frameworks developed from this project will be able to assist educators to develop curriculum and pedagogy, and classroom practices and learning environments that foster increased academic engagement and intrinsic motivation in a social studies classroom and beyond.

## Significance of the Study

Academic engagement decreases significantly from the early grades of elementary to high school. One reason for disengagement is that adolescents do not fully appreciate the value of academic achievement and successful schooling experiences (Eccles et al., 1998; Marks, 2000). Many high school students abandon their sense of wonder when they step into their history, psychology, civics, or philosophy classrooms. They often stop asking questions, make fewer connections with their prior learning and personal experiences, and fail to find meaning. Although teachers work extraordinarily hard to provide their students with the practical uses and meaningfulness of their lessons, and use various teaching strategies to motivate and engage their students to participate in class activities, young students still tune out and disengage from their studies (Toshalis and Nakkula, 2012). Figuring out what motivates and engages high school students is a critical question that needs to be addressed.

Although there has been a growing awareness of the significance of adolescents’ engagement in education, there has been little scholarship documenting p4cHI or PI’s effects on student learning engagement and motivation. To fill the void, this research examines how the PI course and p4cHI approach to education promote involvement of economically disadvantaged youth in academic engagement.

Adolescence is a critical psychological stage between childhood and adulthood that deserves particular attention. The onset of adolescence is a time of rapid physical growth, sexual maturation, and social and emotional changes, and it also implies a wide range of behavioral and emotional health problems. Pubertal development and brain maturation shape adolescent development and presumably later behavior (Erikson, 1963; Board of Children Youth and Families, 2004; Forbes and Dahl, 2010). This work will extend adolescent research to students’ academic engagement in the association of PI. The investigation and description of various factors that contribute to adolescents’ academic engagement in the PI course from the perspective of students themselves will provide educators, researchers, and policy makers with important insights into the practicalities of PI course design, revision, and implementation, especially in regard of the complex educational and psychological development of adolescents.

## Research Questions

The major research question driving this study is: In what ways does the PI course influence high school students’ learning engagement? Do students feel more engaged in their learning during and after taking the PI course? If so, what reasons for this do they report?

## Theoretical Framework

Vygotskian and Deweyan educational theories are used to support this study. It informs the research questions, methodology, and data analysis of this study. Over the last 30 years, research on p4cHI has been viewed through a variety of theoretical perspectives: multicultural educational theory, social constructivism learning theories, learner-centered ideology, identity exploration theories (Makaiau, 2010, 2013), constructive grounded theory (Miller, 2013), social cognitive theory, community of inquiry theory (Jones, 2012), and Deweyan and Vygotskyian theories (Bleazby, 2007; Makaiau and Lukey, 2013). Those previous studies provide the rationale for the selection of Dewey’s theory of education and Vygotsky’s social constructivism theory for this study.

Graham et al. (2007) argued, “the idea that students must be actively engaged in the learning process in order for it to be effective is not new. The roots for active learning reach back in the literature to John Dewey” (p. 233). In the 1930s, Dewey proposed the radical transformation of schools that contributed to the creation of career and technical education courses in order to promote student engagement (Fletcher, 2020). According to Dewey, first, academic achievement is positively influenced by the amount of active and collaborative *participation* in the learning process (Dewey, 1997; Coates, 2007). Second, authentic interest can be best achieved when teachers are able to find the students *preferences, needs, and skills* in the subject matter. The planning and teaching, studies, and topics included in the course of study should enrich students’ *lives* and consider their *direct interest*. Third, one way to reinvigorate schooling is to make more use of students’ *out-of-school experiences*, as they are more likely to encourage reflection. Engagement occurs when students engage in activities related to their interests and competence (Lam, 2013).

In the context of the KHS PI social studies curriculum, students actively engage in their discussion and take the major responsibility for their learning. Learning by doing, or the incorporation of activity and experience in the classrooms, is at the heart of PI class. PI students can “grow in their own natural self-actualizing ways” rather than get trained by imposed knowledge and skills (Schiro, 2008, p. 98). The goals of the PI class are to integrate students’ experiences, consider their interests, support active participation, deepen their thinking, and encourage multiple opinions, which are closely connected with Dewey’s educational philosophy.

Vygotsky’s (1962) sociocultural theory also develops a construct of academic engagement in classrooms. First, academic engagement requires *intellectual* and *affective involvement*. Students’ cognitive development occurs with social, emotional, and motivational investment during activities. Second, a social constructivist classroom is a highly *literate place* where students and teachers can exchange ideas effectively. Third, the activities designed in the classrooms, no matter if it is reading or writing, are *shared socially* (Palincsar, 1998). When students participate in challenging activities, the more capable peers and teachers will guide and support the learners’ learning and thinking. *Social interaction* thus plays a fundamental and inseparable role in the process of cognitive development (Oakes and Lipton, 1999). Fourth, since *environmental factors* affect students’ learning experiences, it is necessary to create a safe and supportive environment in the classroom.

The main Vygotskian theory at work in the PI classroom is the idea that a student’s cultural development appears in two levels. First, they raise their own questions in the individual level. Then, they vote and discuss the questions on the social level. Lastly, they internalize the new knowledge and reconstruct their understandings from *interpsychological* to *intrapsychological* level (Cam, 2006). As Philip Cam (2006) writes, “it would be a natural extension of Vygotskian psychology to suggest that children come to think for themselves through the internalization of social practices” (p. 45). In conclusion, Dewey and Vygotsky’s theories provide scholars and practitioners with a common language and a frame of references for understanding this research.

## Literature Review

Over 30 years of U.S. and international research, including recent studies done in Hawaii, indicate that the use of PI with a group of students who are supported by trusted facilitators and peers to interact respectfully and critically as they explore intellectually challenging questions, known as an intellectually safe community of inquiry, sharpens students’ abilities to “think for themselves” (Lipman et al., 1980, p.53). This activity also positively affects students’ cognitive and social–affective abilities, engagement, moral dispositions, and self-confidence (Lukey, 2004; Jones, 2012; Toyoda, 2012; Yos, 2012; Makaiau, 2013). Even so, there has been very little written about the intersection between students’ academic engagement and p4cHI in the education of adolescents at a Hawaii public high school. The following section will introduce the meaning of engagement and demonstrate p4cHI researches on adolescents’ academic engagement.

### Academic Engagement

Engagement is “the student’s psychological investment in and effort directed toward learning, understanding, or mastering the knowledge, skills, or crafts that academic work is intended to promote.” Students show engagement by seeking out activities and displaying their curiosity, a desire to learn, and positive emotional responses to the process of learning (Newmann, 1992). Authentic, meaningful engagement, though observable, is an internal action. Zyngier (2008) reviews the psychological definition of engagement as a combination of student behaviors, emotions, and cognitive abilities: Psychological definitions are commonly a mix of (i) *behavioral* aspects of the student as doing the work, following the rules, persisting, and participating, while (ii) the *emotional* aspects center interest, value, and feelings (negative and positive) toward the school, the class, and the teacher, and (iii) *cognitive* engagement (psychological investment) includes motivation, effort, and strategy use of students. These views see student engagement as something students *do* and that teachers can *organize* for them (p. 1769).

This means that in addition to being interested in the academic needs of the students, teachers are deeply concerned with the social, emotional, cognitive, behavioral, and physical state of learners. Teachers are acutely aware of the emotional aspects of learning (Goleman, 1995) and design classroom practices that cultivate the making of meaningful relationships. Learner-centered teachers view building relationships of care and trust as a prerequisite to academic engagement (Bluestein, 2001), including higher levels of cognitive thinking (Noddings, 1992, 2002). However, it is often believed that schools tend to be impersonal spaces that fail to individually and personally engage students (Kohn, 2004). Often, they become “institutions of isolation” (Delpit, 2006, p. 179) that discourage individual development.

The National Research Council published a comprehensive study concerning the lack of engagement in today’s public high schools. Many of the students who are retained at schools attend irregularly, exert modest effort on schoolwork, and learn little. This situation can be changed if schools “help the young make sense of life, of experience, and of an unknowable future” (Brady, 2006, p.47). Students are more likely to show both short- and long-term commitment to learning if the class activities are consistently personally relevant, enjoyable, and appropriately challenging (Csikszentmihalyi et al., 1993; Csikszentmihalyi and Schneider, 2000). When students learn subjects that they are interested in and have autonomy in making choices, they tend to perform better (Pintrick and Schunk, 2002; Stipek, 2002). If students pursue an activity out of genuine interest, their commitment will be both more persistent and more successful than those who do not (Armes, 1992). Research has shown that the more educators give their students choice, control, challenge, and opportunities for collaboration, the more their motivation and engagement are likely to rise (Toshalis and Nakkula, 2012).

Three empirical studies on adolescents’ learning, motivation, and reaction to the p4cHI were conducted in the past 10 years. Miller’s (2013) research showed that an overwhelming majority of KHS students thought school had no meaningful connection to their lives outside of school. They believed that school was boring and disconnected, but it is necessary to go to college and “make a lot of money.” While Miller integrated p4cHI in his English curriculum, students started to personally construct meaning through the practice and improvement of their thinking and reasoning. They were able to discuss and weigh ideas about philosophical issues and contents beyond the English texts. More significantly, the students not only recognized their intellectual growth but also took ownership of their learning process. Jones’ (2012) study found that the implementation of a student-centered curriculum that utilizes the p4cHI approach improved student cognitive, social, and emotional engagement, especially student’s perception of self as a learner. The findings of this study revealed that there is a strong connection between the level of student personal engagement and student academic and personal success. From 2005 to 2007, Makaiau (2010) worked together with the Asian/Pacific Islander Youth Violence Prevention Center at the University of Hawaii at Manoa to conduct a large−scale qualitative study that involved 89 KHS ethnic studies students. The study found that applying PI in the course, students not only appeared to grow academically but also personally and interpersonally. Academically, the students developed their abilities to construct philosophical questions, gather relevant information for an inquiry from a variety of sources, analyze data, construct a well−reasoned thesis, write, reflect, and participate in a philosophical community of inquiry.

### Philosophy for Children Hawaii (p4cHI)

Since the PI course is grounded in the theory and practice of P4C, it is important to introduce the conception of P4C in this part.

Philosophy for Children began around 1969 when Matthew Lipman (1993, 2003), a Columbia University philosophy professor, became disenchanted with the educational system. He observed that children did not think as well as they could or should in a democratic society. He observed and was concerned that schools encouraged children to have a negative view of their own intellectual abilities. To address these issues, Lipman created a curriculum that incorporated the skills of logic and reasoning found in the practice of philosophy to improve students’ thinking in the K–12 setting. In an effort to extend Lipman’s original curriculum and vision to a variety of geocultural contexts, a number of P4C Centers have been established worldwide. The Uehiro Academy for Philosophy and Ethics in Education is one of them, which was located at the University of Hawaii at Manoa and was established by the initiator of p4cHI movement, Dr. Thomas Jackson (2012, 2013). Jackson and his colleagues are cultivating a K–12 philosophical schooling experience that encourages students to think collaboratively about meaningful topics and questions that arise from their interests, experiences, and learning contexts.

Jackson’s p4cHI has been adapted and expanded Lipman’s original P4C to serve the various populations in Hawaiian Islands. It provided a more flexible approach than Lipman’s P4C, whose P4C emphasized to incorporate the skills of logic and reasoning found in the practice of philosophy to improve students’ thinking (Miller, 2013). Jackson (2017) branched his viewpoints of bringing the primal wonderment of philosophy from opportunities to move away from Lipman’s novel and teacher manuals to put more emphasis on the building of *an intellectually safe community* influenced by the Aloha culture, the *“little p” philosophy*, the activity of coinquiry between the teacher and students, the *context and content sensitive* (Makaiau, 2010) learning experiences, and *self-corrective reflection*. In Jackson’s (2017) words, his p4c Hawaii views

philosophical activity as grounded in inquiry, not argument, and to view our content as arising from the interests of the community, highly sensitive to the culture and norms of that community, as well as, in some classroom contexts, discipline specific content such as science, math, language, arts, and social studies. (p. 33)

#### An Intellectually Safe Community

The concept of *intellectual safety* is the most important feature of p4cHI approach to education. Jackson (2001) states:

In an intellectually safe place there are no put-downs and no comments intended to belittle, negate, devalue, or ridicule. Within this place, the groups accept virtually any question or comment, so long as it is respectful of the other members of the circle. What develops is a growing trust among the participants and with it the courage to present one’s own thoughts, however tentative initially, on complex and difficult issues. (p. 460)

The p4cHI way of building up a community includes an application of the Hawaiian spirit of *aloha*. Aloha in the Hawaiian language means affection, love, peace, compassion, mercy, goodbye, and hello, among other sentiments of a similar nature. It is this spirit that students can mediate multicultural tensions and build a sense of community between diverse groups of people in the islands (Makaiau, 2017). It is also this sense of intellectual safety that makes participants’ interests, cultures, languages, histories, socioeconomic backgrounds, and other aspects of their identities are included and validated during the community development and serves as a basic foundation for PI (Makaiau et al., 2017).

#### “little p” Philosophy and p4c Inquiry

Agreeing with Plato and Aristotle, Jackson (2004) believed that philosophy begins in wonder. However, he also argued that in the classroom, philosophical thinking associated with wonder did not need to be based solely on the Western academic perspective of philosophy, which he called “Big P” philosophy, such as metaphysics, epistemology, ethics, and practice, that is grounded in the Western model of argument such as reasons, premises, and conclusions. There was also what Jackson (2017) called *“little p”* philosophy that stems from the wonder, questions, and thinking of the students with which we all begin our life. Thus, the two important particular features of p4cHI inquiry are *the inquiry arises out of the interests of the students and begins where students are in their understanding*. Because of the “little p,” the P4C Hawaii is abbreviated as p4cHI. It enables the students to “properly, rightly, compassionately participate in our diverse worlds with the rich varieties of sounds and actions of those around us” (p. 35).

#### Content- and Context-Sensitive p4c Hawaii

Although, in the beginning, the Hawaiian P4C practitioners used Lipman’s theories and concepts, soon, they found that Lipman’s model of using specific novels and his version of P4C made teachers very difficult to teach content-specific classes in regular classroom practice. It was not easy for teachers to move from the text to “Leading Ideas” to the use of “Exercises” and “Discussion Plans” provided in the manuals. Thus, they adopted a more concretely designed and flexibly implemented p4cHI approach, responding to Lipman’s insightful analysis of critical thinking, the “context sensitivity.” This approach takes the stance that philosophy is an instructive element of classroom pedagogy and a way of responding to “content” that begins with the questions of the students while it is sensitive to the content being taught and the cultural context of the learning environment (Makaiau and Miller, 2012).

#### The Good Thinker’s Tool Kit

A p4cHI approach to education encourages teachers and students to brainstorm, implement, and reflect on new ways of incorporating *community, inquiry, philosophy*, and *reflection* into a wide array of subject areas and diverse community contexts. It is based on a set of teaching strategies that can guide teachers to translate those theoretical foundations into classroom practices (Jackson, 2012, p. 6). The entire process of the Plain Vanilla^1^, the posing of questions using the Good Thinker’s Toolkit, the use of the Community Ball, a sharing of different perspectives including that of the instructor as a coinquirer, and reflecting and evaluating at the end, provides a concrete procedure to transform philosophy and thinking into real classroom practice. The Good Thinker’s Tool Kit consists of seven indicators for critical thinking, which is an essential component of the Kailua students’ p4cHI practice.

W—What do you mean by that?
R—What are the reasons?
A—What is being assumed? Or what can I assume?
I—Can I infer ___ from ___? Or where are there inferences made?
T—Is what is being said true and what does it imply if it is true?
E—Are there any examples to prove what is being said?
C—Are there any counter-examples to disprove what is being said?

A considerable number of empirical studies into the effects of P4C have been conducted (Sutcliffe, 2003; Trickey and Topping, 2004, 2006, 2007; Garcia-Moriyon et al., 2005; Topping and Trickey, 2007), and they have produced strong support for the practice of P4C or philosophical community of inquiry, in terms of cognitive, social, and emotional benefits. Yet many of the studies have been more focused on reading, critical thinking, and mathematical abilities than on academic engagement, social, and affective benefits. Additional rigorous studies are needed to examine the psychological benefits of using p4cHI in the classroom.

## Materials and Methods

This case study is “interested in uncovering the meaning of a phenomenon” for the PI participants (Merriam, 2009, p. 5). The phenomenon in question is participants’ learning engagement in the PI classroom. Do students report feeling more engaged in their learning through p4cHI, and if so, what are the reasons they attribute to this?

### Setting: Kailua High School

Kailua High School was founded in 1955 and was moved to its present location in 1962. With its beautiful views of the Ko‘olau mountain range, KHS is one of four public high schools that serve the Windward (eastern side) District on Oahu. The rural communities of Kailua and Waimanalo each provide about 50% of the population of just under 1000 students at KHS (2014 total enrollment = 750), among those just under 60% of the students are native Hawaiian. As more than 40% of the student population comes from low-income families, KHS receives Title I funding. Many students are faced with domestic violence, discrimination, and substance abuse (Makaiau, 2010). The school utilizes programs such as p4cHI and Habits of Mind to prepare mindful, philosophical thinkers who will pursue their life goals and create positive changes in the world (Kailua High School, 2013).

### Participants

Students were recruited from the PI course at KHS in Fall, 2014. Pseudonyms are used to protect the identities of the four girls and two boys. Their ages range from 15 to 17. Five of them are mixed race, and one is Japanese ethnicity.

### Data Sources

This multiple case study uses multiple sources of evidence. The following documents help “uncover meaning, develop understanding, and discover insights relevant to the research problem” (Merriam, 2009, p. 163).

#### Class Discussion

The PI class ran for 8 weeks. Five classes were held each week, for 65 min each in the late mornings, except on Wednesdays when class was only 45 min for a total of 35 classes. Students engaged in philosophical inquiries using the Good Thinker’s Toolkit and Plain Vanilla (Jackson, 2013) on topics such as racial politics (i.e., race and ethnicity in Hawai‘i; what if there were no governments), and gender and society (i.e., Bel Hooks—feminism is for everyone; what is it like to be somebody else). Twenty student class discussions (CDs) out of 35 classes were recorded. The full length of the video-recordings is about 21 h.

#### Student Work

Besides CD, additional student work was collected throughout the semester including student handwritten responses in class to a set of open-ended questions or sheets provided to them in the workbook named The Daily Record, PI Student Resources, and Workspace (Makaiau et al., 2014). The following section will describe student work in detail.

##### Philosophical inquiry daily reflection

Students used the PI daily reflection (DR) in every class. In the DR, they reflected on the prompt of the day (POD), which was a quote, a short video, a song, a poem, or movie related to the class. They needed to “use textual evidence and/or self-knowledge/experiences to support” their responses (p. 63).

##### Philosophical insight paper

Students used philosophical insight paper (PIP) to continue thinking about the topic they philosophized about after each unit (e.g., what is the meaning of life? Am I the same person that I used to be?). The PIP was organized into five sections: (a) Evaluation of the Community of Inquiry; (b) Lenses of PI; (c) Constructed Response using Claims, Assumptions, Supporting Evidence, and Counter-example; (d) Personal Reflection and Action; and (e) References (p. 225). At the end of the semester, each participant submitted two PIPs.

##### Inquiry memos

During each Plain Vanilla discussion, students used inquiry memos to record their questions and thoughts, as well as those of their peers. The inquiry memo data were collected after each Plain Vanilla discussion.

##### Final take-home reflection paper

In the final take-home reflection paper (FRP), students reflected on their experiences in the PI course at the end of the semester.

#### Focus Group Interview

A follow-up focus group regarding student academic engagement with four PI participants (originally there were six participants, but two of them did not continue in the class after mid-term) using a semistructured interview approach (Merriam, 2009) was conducted by the end of the semester.

### Data Analysis

Data from student written work, classroom discussions, and field notes were analyzed, as they were collected. While organizing and analyzing data, NVivo software, Mac trial version, was used.

Analysis of qualitative data occurred in three phases. In phase 1, all qualitative data were entered into the NVivo software, and initial open codes were developed to highlight major themes occurring in each individual case study. The analysis made use of all of the relevant evidences, considered major rival interpretations, and addressed the most significant aspects of each case study. Salient themes that appeared in each individual case study are reported in the format of concept maps and narratives based on the occurrence frequency that was shown in the NVivo software.

In phase 2, using the method of constant comparison (Strauss and Corbin, 1998; Merriam, 2009), similarities, differences, and complementarities across and within participants were examined in a cross-case study analysis. The six cases were studied collectively in order to inquire into similarities and differences in students’ learning engagement (Denzin and Lincoln, 1998). A categorical analysis strategy was utilized to break down the narrative data and rearrange those data to produce bigger categories that facilitated comparisons. In order to provide intuitive data analysis results, flow charts were created to tabulate frequency of themes. Concept maps were used to categorize and recombine data.

During phase 3, three types of qualitative data, the CD, daily written reflections, and observation notes; focus group interview; and PIP and final reflection paper were triangulated in order to increase the trustworthiness of the study. The final themes were refined and reread with critical friends.

## Results

This multiple case study consists of six individual case studies of students in the PI class (see Table 1). Each student has an individual perspective about an engaging PI classroom that is anchored in his or her life context, but there were several common themes emerged in the cross-case analysis. Based on the frequency count in the NVivo software, the reasons that six participants felt engaged in learning in the PI course are (a) the PI class created an intellectually safe environment that fostered students’ learning and development; (b) participants inquired together into the topics and questions that they are really interested; and (c) participating in communities of philosophical inquiries broadened their understandings of themselves and others. Besides that, listening attentively and carefully to their peers and teacher’s ideas benefited both themselves and others. Building up a strong community helped them engage in their learning as well. Each participant has their own understanding of an engaged PI classroom. Kalani appreciated how an intellectually safe environment promoted his learning experiences.

**TABLE 1:** PI participant demographics.

Participants	Gender	Age	Grade	Ethnicity
Kalani	Male	17	12th	Hawaiian, Portuguese, Chinese, Japanese
Nahele	Male	17	12th	Caucasian, Japanese
Peleke	Male	16	11th	Chinese, Caucasian, Part-Hawaiian
Liko	Female	16	11th	Japanese
Makali	Male	15	10th	Caucasian, Japanese, German
Kanani	Female	17	12th	Hawaiian, Chinese

Not being scared or worried that others will judge you. You can express your thoughts or feelings with others who will support you and listen to you. It’s a feeling like being with someone you trust or like being with your family who listen to you and hold you up. I see this class as a family and I know I can express myself freely. (CD, 10/16/2014)

Nahele shared in the focus group that he was more engaged in learning in the PI course. The most important reason is that the PI course was interesting, and he could be able to say how they feel about the subjects they learned. For instance, he expressed that:

I think we are more engaged [without doubt]. Because in other social studies classes, you are not allowed to say how you feel about certain things, you just learn it, you are supposed to read about it, and just accept what you read. While in this class…you get to make connections to your life, you get to listen to other people’s saying. (FG, 12/18)

Nahele commented after the PI course, he still wondered about the topics discussed in the classroom. He was motivated to come back to the classroom to talk about it again. Peleke believed that communications made learning engaging. Liko thought she was more motivated to learn in the PI course: “I think this class motivates me by like I am never challenged to think outside my own thinking, so yeah [I like this class].” She felt that History was boring, but in the PI course, she could “get to reflect how we feel about it [subjects]” (DR, 12/18). Makali liked the PI course, “because there are actual community” (DR, 10/30). He used a counterexample to describe a class he disliked: “In ethnic studies, last year I didn’t know anyone and I hated that class” (DR, 10/20). Kanani’s data indicate that peer acknowledgment and support had a strong positive impact on her academic engagement. She appreciated that the PI course inspired to her to think outside of the box.

**FIGURE 1:**
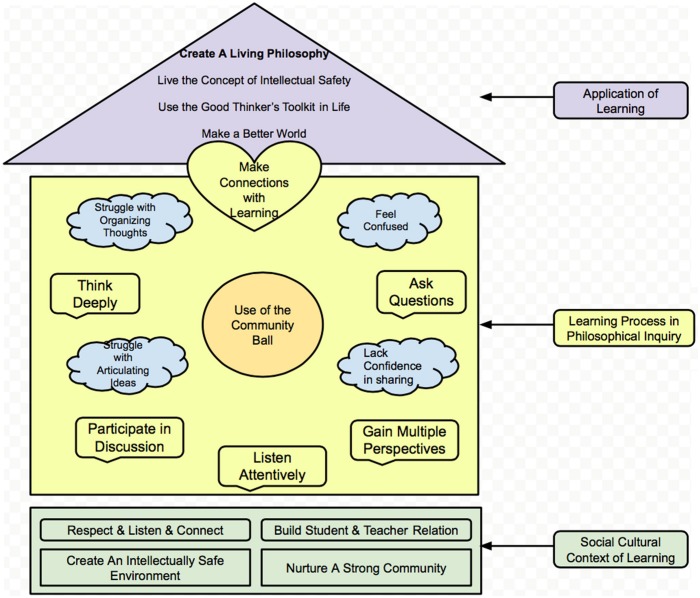
The philosophical inquiry student academic engagement framework.

Based on the cross-case analysis, a conceptual framework of student perceptions of academic engagement in the PI class is presented in Figure 1. This was created based on each student’s salient themes counted in the NVivo software. In general, the six participants’ perceptions of an engaging PI classroom can be categorized into three main themes, which correspond to the three parts of this conceptual framework of a house.

### Social Cultural Context of Learning

Maintaining a safe and positive classroom environment is a fundamental condition for learning. In the conceptual framework, this part is colored in green, representing that a positive classroom culture creates a nurturing foundation for learning. The intellectually safe environment developed a constructive, creative, and methodological culture of thinking and communication. For example, Kalani shared in the classroom that it was his own responsibility to maintain an intellectual safe environment. The intellectual safety set a foundation for a supportive and collaborative learning environment. Kalani explained that in the PI course, “No one was really putted down or felt unsafe, everyone gets along while in the discussion” (CD, 11/6). Peleke realized that even though he had a disagreement or argument against an idea or one person, he could still examine its benefits. He learned to be open-minded and think critically.

In the PI course, the students and teacher cocreated a social–cultural learning context that ensured a deep PI could occur. Prior to the PI class, the students and teacher coconstructed a definition of intellectual safety and made a community ball to facilitate their turn taking. While making the community ball, they began to know each other personally. Participants considered the community ball as a tool that helped them to manage the classroom and engage in coinquiry with their teacher. According to Nahele, the uniqueness of the community ball was that students became respectful and attentive while sharing thoughts. “Because of this, not only does it represent our community, but it also represents the power to speak so that during inquiry, each person who receives the ball is allowed to express their opinion hopefully without interruption” (DR, 10/20).

While engaging in a number of reflective activities and readings that reiterated the importance of intellectual safety and community building, the students began to build up a strong community and a good relationship with their teacher. For instance, the biggest takeaway for Kalani was “just making a bond, and making a strong community” (FG, 12/18). Like Kalani, Nahele appreciated that the class participants built up a strong community that encouraged the gifts and strengths of every participant and promoted a sense of belonging and purpose. He suggested that his peers “strengthen the community further…. we can keep it up. So it’s not boring” (CD, 10/21).

Living the concept of intellectual safety, students transformed their learning into an art of democracy. They respected each other’s ideas, interests, and needs. They listened attentively to what others had to say, and shared their thoughts genuinely. They were continuously working on cultivating and nurturing a sense of belongingness and connectedness in and out of the class. This social context of learning sets a psychological foundation for students’ further learning in the PI.

### Learning Process in Philosophical Inquiry

The learning process in the concept model is colored in yellow, representing the “aha” and mind “sparkling” moments that students experienced. Because the community ball is a symbol of empowerment, it is painted an orange color. The challenges take the shape of a cloud, which means that although the students experienced confusion and struggles, these could nurture new realizations. These activities are in the living area of the house model, representing the daily work of learning and realizing.

The PI class worked to create a learning environment that maximized each learner’s ability to interact with each other, especially with the teacher. Kalani expressed his gratitude to his teacher and described that, “I feel good [studying in this classroom]. I love her [the teacher]” (CD, 12/19). Nahele reported that he built a better social relationship with his teacher, and described,

I guess I feel afraid my teachers in a social level. Because you just walk in the class, you tell them, they just tell you this this, and then you go home, you don’t talk them. Miss Shiroma is like, I don’t know, you kind of like on a social level, because we know how she thinks and feels about certain topics. And I think it really helps with the whole community building thing. (12/18)

Students were seated in a circle and engaged in PI through social interaction and communication. The class puts a premium on students’ inner interests and needs, so students were able to raise questions that they genuinely wondered about. Although there was not always a definitive answer to each question, students were eager to explore the solutions and think alongside each other, appreciating peers who were more able to articulate ideas and explain thoughts. Taking Nahele as a typical example, he explained, “It’s good to ask questions and strive to ask more, but even better if everyone make an attempt to answer them” (CD, 11/6). He enjoyed using Good Thinker’s Tool Kit to ask questions and used it beyond the classroom. He commented, “Within our community of inquiry, we get more by giving to expand on our discussion” (DR, 10/29). As a fortuitous byproduct of this newfound expanded perception, Nahele was more engaged in his thinking. He demonstrated new connections with his learning as he questioned: “Why does racism exist? Where did racism originate from?” (DR, 12/10). He showed insight into his own thinking when he said, “I’m taking away that maybe everything we do is subconsciously selfish, even if to the smallest degree. Is it purely for other people? Does altruism really exist?” (DR, 10/24) On another day, he explored ideas around morality. “We teach children certain rules of morality growing up. At what point do they become irrelevant in our lives? What are the reasons we disregard them in life?” (DR, 12/18). These internal dialogs indicated that the student had internalized good reasoning skills and were learning to think for themselves.

Using the community ball to issue the invitation, students were empowered to share their personal stories, challenges, raw thoughts, and not clearly formulated ideas. Students enjoyed the academic freedom to explore meaningful and controversial issues that arose from their life and context. Peleke thought he actually was rewarded with more knowledge and more strength. He positively commented that, “From that it actually helps yourself and other people, you are not being one sided, relying that one information” (CD, 10/30). Their discussions were connected with their prior experiences, thoughts, feelings, and ideas, and learned through these experiences in the classroom. Because of this encouraging and safe community of learners, Liko was able to overcome her experiences of insults in other classes.

The PI participants were sometimes confused by their own questions and by those of others during their discussions. They experienced challenges in organizing their thoughts and articulating their ideas. Peleke shared,

I am walking away with that I need to try to understand more terms. I need to make people understand what I am saying. I just want to have clarification, examples, probably I have to look up some new words to understand and to figure out. (CD, 11/6)

Some students initially lacked confidence in sharing their thoughts. For instance, Kanani exhibited low self-efficacy in her thinking. “I wrote it, but…because I…I wrote it, I don’t think it’s a good reflection. I don’t understand” (CD, 10/16). She felt it was a risk to share her ideas in the classroom. She explained,

“I said we need to take certain risk in order for us to move forward. When I didn’t wanna share, I think this is the first day in our class, I didn’t wanna share, but then I also feel I have to take the risk in sharing in order for our task to move on. (CD, 11/17)

Kanani had a unique challenge that other participants did not share in the PI course, which was that she had to work almost 10 h each day instead of focusing on studies. She appreciated that the PI course inspired to her to think outside of the box. Yet she also experienced many challenges she could not handle during the course, such as articulating her ideas and thinking deeply. All these challenges may result in Kanani’s absence from school.

By the end of the semester, students all learned certain reasoning skills (i.e., to raise questions, to make assumptions, to use evidences, to apply the Good Thinker’s Tool Kit), as well as to make decisions and solve problems. Students expressed that they appreciated the multiple perspectives gained from their peers, teachers, and guests because they developed an understanding of ideas from a range of areas and obtained the skills, knowledge, and attitude to interpret these ideas and to live their lives better.

### Application of Learning

When engaging in discussions, participants were exposed to multiple perspectives, which inspired them to reflect on their own thinking, examine personal beliefs, and then make changes in their lives. The class awakened students’ inner selves and helped them realize their own unique potentials. Peleke increased his confidence in expressing personal beliefs. For example, he wrote,

I am starting a personal change from taking this course due to the interaction and participation in what p4c feels on a daily basis and also I have become less anxious due to me participating within in the community and sharing my opinions and ideas on the work we are given and at times on my own personal life. (12/18)

They began to think about the purpose and meaning of their lives. Each student actively chose his or her own way to construct the meaning of his or her particular life. They created a living philosophy and applied new learning in how they made decisions and lived their lives. For example, they engaged in PI with friends, and brought the concept of intellectual safety to their family and community. For instance, Makali was involved in p4cHI activities outside of the classroom.

I would say that this was the fun of this class in order to be able to take this outside.… my friend…started to really use all the terms, like what are the reasons, can I assume…. We ended up having this kind of discussions after lunch after school. It’s really interesting….That makes me think deeply about anything. (CD, 11/6)

The PI participants not only took into account their own inclinations and options for a meaningful life but also took into consideration the need for a more humane and democratic society. They started to build a more holistic and integral understanding of themselves and the society. They learned to put their engaging and dynamic reflections into practice. For instance, after learning ethical egoism and altruism, Liko asked, “What I realized is that talking about benefiting ourselves. We talked about having good or bad intentions. I thought, what makes you have these good or bad intentions, and why you act upon them?” (CD, 10/17) Liko began to question human nature and her self-knowledge; she asked, “When I heard everyone, I have more values. What I think is right? What makes me happier? What would I think human nature is?” (CD, 10/28) She also thought about “What is morally right and wrong?” (DR, 12/5) She related her learning to the world problems, and questioned, “Is there really a way to get rid of racism? WATRs [What are the reasons] why we can’t get rid of racism?” (DR, 12/10) She showed her care to the environment, and asked “WATRs [What are the reasons] people are so cruel to the environment?” (DR, 12/16)

They were interested in personal happiness and wanted to lead balanced and peaceful lives. They were inspired to strive for ideals of social justice, democracy, and multiculturalism, and to contribute to the public good. In class, they interrogated the social, political, economical, and moral imperatives of society, which helped them through the developmental transition period of adolescence. They discovered the hidden voices of women, children, minorities, nature, and of those who are marginalized. Many of them continued to think about the questions posed in class when they went back home and looked forward to coming back to this class again. These characteristics are placed just under the roof of the house, the highest place. The roof is shaped like a triangle, similar to Maslow’s (1968) hierarchy of needs. These skills and purposes will hopefully help students to develop increased self-esteem and self-actualization. This is also one goal of education, making students use the new knowledge and resources around them, and helping them transcend their thinking and living. The color is purple, commemorating royalty, or the best in each of us.

## Discussion

Applying qualitative methods, this study developed a deeper understanding of what classroom contexts, conditions, discourses, tools, and practices promote adolescent learning experience. Referring to the PI Student Academic Engagement Framework (Figure 1), the six participants’ perceptions of an engaging PI classroom can be categorized into three main themes: First, maintaining a safe and positive classroom environment is a fundamental condition for learning. Second, asking questions, sharing ideas, listening attentively, thinking deeply, and making connections are the manifestations of an engaging classroom in the PI process. Third, students transcend their learning experiences by living a new philosophy.

In the student qualitative data, the most important reason for students to engage in learning, or the most salient theme that appeared, was that the PI class created an intellectually safe environment that fostered students’ learning and development. Echoing Vygotsky (1935/1994)’s theory, the context of a social–historical environment can significantly influence students’ learning. Maintaining a positive classroom environment is a fundamental condition for students to thrive in learning. Each individual is a socially grounded self, and is “in the ongoing process of living in a social environment” (Campbell, 1995, p. 40). It is necessary for students and teachers to create an intellectually safe environment in the classroom. This understanding gives way to a respect for opposing viewpoints and claims that often arise out of an inquiry. Rather than having differences divert the direction of the inquiry into an argument or popularity contest, an intellectually safe class will recognize, examine, and celebrate them. This awareness is necessary to create a less intimidating classroom environment, allowing for all students to be active contributors to the community of inquiry process.

Participants indicated that the circular seating format and smaller class size helped establish an environment that could free them from some social and emotional stresses. The intellectually safe community provided them with ongoing opportunities to build up connections and relationships among each other. The removal of judgment and fear created a space where, despite their different ethnic backgrounds, beliefs, and worldviews, they could openly share their personal experiences and explore controversial issues with their peers (Makaiau, 2010; Miller, 2013). In the PI classroom, Nahele and Makali often raised their voices and frequently volunteered to contribute. Kalani and Peleke were more reflective learners who typically developed ideas and questions in their minds before speaking. Liko was a shy student who felt uncomfortable speaking in front of groups, at least initially in the first week of the class. Kanani was not confident in sharing her ideas. These differences may be due to learning preferences as well as personalities. However, a strong community enabled Liko and Kanani and other students with different learning styles and personalities to contribute. Active participants such as Nahele and Makali were able to use the community ball to invite Liko and Kanani to share.

Research showed that “persons with significant difficulties relating to others interpersonally often have related academic struggles in the classroom particularly as they get older” (Winner, 2011, p. 4). Freire (1970) wrote, “Education must begin with the solution of the teacher–student contradiction, by reconciling the poles of the contradiction so that both are simultaneously teachers and students” (p. 72). The teacher added an important dimension to students’ social relationships. Teachers were often the more capable peers (Vygotsky, 1978) in the classroom, especially in the beginning that pushed students to think deeper and broader. Teachers are not only facilitators through asking questions such as, “What do you mean by…” or “Could you use a specific example to explain…,” but also participants by contributing their own thinking and ideas to the inquiry. As a coparticipant, teachers become “real” with their students, so an atmosphere of trust is built (Purkey and Novak, 1996, p. 50). As the community matures, the role between teachers and students began to blur, as students’ opinions increasingly influenced their teacher or changed their teacher’s thinking. Through social interaction, participants, teachers, and students actively created, interpreted, reorganized, and reconstructed knowledge in individual and meaningful ways. The fundamental norms and culture of a classroom were transformed because the PI classroom has achieved a new pattern of teacher-and-student relationship and interaction, making students and teachers more connected.

Described in the foundation part of the house model, when students’ basic psychological needs of safety, belongingness, and esteem are satisfied in the classroom, they developed better socially and cognitively in the PI class (Maslow, 1968, 1987). In the p4cHI PI process, participants thought and inquired alongside their peers and their teacher into the topics and questions that they genuinely wondered about. Participants criticized other social studies classes, like history, where they had to memorize facts and events. Knowing students’ concerns and motives, the PI course was designed to include, but not limit, and to integrate students’ experiences and prior knowledge, consider their interests and needs, support active participation and discussion, deepen their thinking and inquiry, and encourage multiple perspectives. The class created opportunities for students to wonder, discover, explore, and imagine and allowed students to experience what that feels like. They simply found their chosen topics and learning interesting.

Student academic achievement is positively influenced by the amount of active and collaborative participation in the learning process (Coates, 2007). The level of engagement and collaboration, and the excitement among the PI participants while engaging in Plain Vanilla activities reshaped their learning into an esthetic experience because it was full of life and its own form of beauty and spontaneity. They transformed from passive participants to active agents of thought and change in their class and life.

## Conclusion

One current crisis in education is that students lack real interpersonal connections. Although contemporary formal education helps students gain tremendous external knowledge, accumulate skills and wealth to become good citizens, and become members of the working force, emphasis on the basics of human life and existence such as health, happiness, and human values is too often overlooked or entirely missing throughout the worldwide educational systems (Ozmon and Craver, 2007). While education and schooling increasingly strive to integrate technology into teaching and learning, high-speed Internet and social communication tools do not seem to strengthen the internal and physical connections among students and communities (Xu, 2013). Younger generations experience this lack of intimacy to a much greater extent. Many adolescents are out of touch with themselves, with others, with nature, with the environment, and with the time they live (Roberts et al., 2009).

Many teachers now cannot fully satisfy students’ psychological and social needs in the class. That’s one reason that students do not feel engaged in their schooling, or cannot even construct meaning that guides and motivates their future development. Deci and Ryan’s (1991, 2008) research summarized that autonomy, competence, and relatedness are the conditions that nurture intrinsic motivation. In the traditional idea of the school, the student’s *personhood* is often ignored; their interests, questions, thoughts, personal experiences, beliefs, and curiosity have been disregarded in the process. Yet in the PI environment, students awakened their spirit to wonder, to question, to explore, and to experiment. In addition to being interested in the academic needs of their students, p4cHI teachers are also deeply concerned with the social, emotional, behavioral, and psychological state of learners. The class moved from the notion that their answers, thoughts, and questions were only “right” if students aligned with those of their teacher or textbook, and that only authority figures had correct answers and would explain “what is text about” and “how to answer this question” once they had finished reading (Miller, 2013). As the students were able to overcome these assumptions and realize that their personal experiences, genuine thoughts, once supported by solid evidences and reason and taken seriously by the teacher and the community, they began to intrinsically engage with their learning.

Imagining a world, wouldn’t it be nice if educators could make classroom environments grounded in our human curiosity for exploration, own enthusiastic desire to construct our own self-defined meaning? The p4cHI community of inquiry creates the space and the opportunity for students to make fundamental connections within their individual selves and with other people. It assists students in making connections in their own thinking, between their emotions and their thinking, and other aspects of their self. It fosters better student-and-teacher connections as they participate in cooperative learning. The students were able to use the thinking tools practiced in the community of inquiry as a way to examine their own lives and frozen thoughts, and challenge their beliefs, which allowed them to create personal significance to the academic content.

The study proved that education would cultivate a better self if we provide students with a time and space to reflect and reconnect within themselves, and with others and the world. Such insights may provide suggestions and implications for teachers to implement more effective P4C education in and beyond the Hawaiian Islands.

## Data Availability Statement

The datasets generated for this study are available on request to the corresponding author.

## Ethics Statement

The studies involving human participants were reviewed and approved by the University of Hawaii at Manoa. Written informed consent to participate in this study was provided by the participants’ legal guardian/next of kin.

## Author Contributions

LL conducted the research and wrote this manuscript.

## Conflict of Interest

The authors declare that the research was conducted in the absence of any commercial or financial relationships that could be construed as a potential conflict of interest.

## Footnotes

1 Plain Vanilla is a strategy for organizing classroom discussion, dialog, and inquiry. It follows five steps: (1) *READ:* Students read or are exposed to some sort of stimulus, such as text, art, music, or video. (2) *QUESTION*: Each student creates a compelling question that was stimulated by the stimulus. (3) *VOTE*: Students vote on a question they want to discuss. (4) *DIALOG, INQUIRY, and DELIBERATION*: During this time, participants are able to provide insights, examples, and counterexamples, and ask questions in order to inquire deeply into the topic behind the question. It is *not* an argument or debate, but considering and exploring multiple perspectives. (5) *REFLECT and EVALUATE*: Closure is created at the end of the inquiry by having each student write or orally share her or his responses to a set of reflective questions.
